# One Function One Tool? A Review on Mutual Exclusivity in Tool Use Learning in Human and Non-human Species

**DOI:** 10.3389/fpsyg.2021.603960

**Published:** 2021-11-23

**Authors:** Thuy Tuong Uyen Tran, Rana Esseily, Dalila Bovet, Ildikó Király

**Affiliations:** ^1^Laboratoire Ethologie Cognition Développement, UPL, Univ Paris Nanterre, Nanterre, France; ^2^MTA-ELTE Social Minds Research Group, Institute of Psychology, ELTE Eötvös Loránd University, Budapest, Hungary; ^3^Department of Cognitive Science, Cognitive Development Center, Central European University, Budapest, Hungary

**Keywords:** mutual exclusivity, social learning, function learning, tool function, functional fixedness, animals, children, evolution

## Abstract

The goal of this review is twofold: first to explore whether mutual exclusivity and functional fixedness overlap and what might be their respective specificities and second, to investigate whether mutual exclusivity as an inferential principle could be applied in other domains than language and whether it can be found in non-human species. In order to do that, we first give an overview of the representative studies of each phenomenon. We then analyze papers on tool use learning in children that studied or observed one of these phenomena. We argue that, despite their common principle -one tool one function- mutual exclusivity and functional fixedness are two distinct phenomena and need to be addressed separately in order to fully understand the mechanisms underlying social learning and cognition. In addition, mutual exclusivity appears to be applicable in other domains than language learning, namely tool use learning and is also found in non-human species when learning symbols and tools.

## Introduction

Learning and cultural transmission is a prominent topic of research, involving a large range of species, from humans to different groups of non-human animals. More specifically, social learning, which is defined as the ability to acquire new skills from observation or interaction with other individuals (Heyes, 1994; Galef and Laland, 2005), has sparked interest among various scientific fields, from psychology to ethology, and even to robotics. Social learning is assumed to be especially important for young and/or novice individuals as they are confronted with an environment that is constantly changing, and thus, they have to adapt quickly by learning new skills where following an expert conspecific offers a shortcut strategy (Hoppitt and Laland, 2013; van der Post et al., 2016). It is also energy- and time-saving, which is not the case for individual learning which also presents risks of predation (Galef and Laland, 2005; Hoppitt and Laland, 2013). As such, social learning has gained much interest because it is believed to play a key role in the development of behavior in social contexts, in the transmission of the socially constituted knowledge of traditions, and thus in helping novices to get acquainted with culture (Tomasello, 1999; Castro and Toro, 2004; Herrmann et al., 2007; Whiten and van Schaik, 2007; Whiten et al., 2009; van Schaik and Burkart, 2011). Indeed, social learning research predominantly has focused on the forms of learning from conspecifics and observable other agents; among the different existing forms, we can find imitation, defined as copying others’ actions (Whiten et al., 2009, see also Whiten, 1998; Horner and Whiten, 2005; Buttelmann et al., 2013) or emulation, defined as a form of learning about the environment or an action’s results (Whiten et al., 2009, see also Horner and Whiten, 2005; Hopper et al., 2008; Tennie et al., 2010). In addition, there is an emerging focus on the underlying mechanism subserving the social learning forms. The objective of grasping the underlying processes responsible for the different forms of social learning allows researchers to turn to the strategies and potential inferential principles that supply such behavior. Thus, this paper contributes to this attempt by focusing on a particular social learning strategy called mutual exclusivity that was extensively studied in linguistics and that has often been confounded with functional fixedness in studies about tool use. Through this review, we examine whether mutual exclusivity is a general learning strategy extending to other domains than language development and whether it is exclusive to humans or if it is shared with other species as well.

Mutual exclusivity is an inferential principle supplying learning from conspecifics that has received significant interest in language acquisition, and recently has been introduced to other domains of learning as well. It originates in the principle of contrast, which states “that any difference in form in a language marks a difference in meaning” (Clark, 1987, p. 1). According to Clark (1987), if the contrast principle is general in language use, it brings about important predictions that are necessarily applicable to expert users and novices, adults and children as well. First, if words contrast in meaning, there are no true synonyms; new words with similar meanings still indicate some differences. In addition, already known words enjoy priority in expression of meaning. Finally, novel words are expected to fill the gaps in the lexicon and to be mapped on a meaning that has not been covered already by a word. These predictions together grant effective communication among the speakers of a common language, by assuming that they tend to use the same words to express similar, specific meanings. Indeed, for children it is essential to presume this principle from a very early age in order to become successful in language acquisition.

Mutual exclusivity is defined as an inferential rule that leads to attributing one exclusive label to an object or object kind (Markman and Wachtel, 1988; Merriman and Bowman, 1989). This strategy allows quick, immediate learning of a new meaning that is called fast mapping (Rice, 1989, p. 152). The use of this strategy has been revealed by a seminal study showing that children between the age of 3 and 5, hearing a novel label, searched for and mapped this label on a new, unfamiliar object; they interpreted the term as a label for the object itself. However, if the only object present was familiar, the name of which was known by them, children rather interpreted the novel label as a part or substance term (Markman and Wachtel, 1988). This strategy in itself allows the speakers learning a distinctive word to share their thoughts and at the same time inferring that the same word will be understood similarly by an addressee. This procedure used in language acquisition and psycholinguistics is similar in structure to how behavior analysts and animal cognition researchers have investigated the emergent learning of new arbitrary associative relations without explicit training, called emergent matching or learning by exclusion (Wilkinson et al., 1998). While emergent matching is a fundamental inference, it has been found to be context sensitive: the success of the inference was modulated by the characteristics of the items to be learned (e.g., it was more difficult for actions than for objects, see Rice et al., 1990); and while in humans it required only few exposure to the defined stimulus sets, in other species it turned out to be dependent on a long familiarization phase (see Schusterman and Kastak, 1993).

Nonetheless, all the above fields argue that emergent matching could be a result of two principles, either by encouraging a search for a novel association, for an undefined item (“new names refer to items that do not yet have a name,” see Golinkoff et al., 1992), or by a process of elimination (rejection of an already named item as a match for the new, undefined label), so by an assumption that a single item cannot have more than one name, and this is the mutual exclusivity principle (Markman, 1989). This very notion of mutual exclusivity has been extended to object function understanding: despite the potential that one object can be used for different purposes, we usually attribute one function as specifically linked to that object (Kelemen, 1999, see also Golinkoff et al., 1992; Diesendruck and Markson, 2001; Casler and Kelemen, 2005, 2007; Petõ et al., 2018). Kelemen (2007) investigated whether function mapping is stable over time, and whether one tool is dedicated to being used for a specific function, shared by a community. In their study, young children chose to use the specific artifact that was used by a model to achieve a particular goal in order to achieve the same outcome. In other words, even toddlers remain faithful to the one dedicated function of a tool they have learned about in a social context, instead of flexibly using any applicable tool to achieve a desired goal (Casler and Kelemen, 2005).

In the domain of tool use, this phenomenon appears to be very similar to another observed behavior pattern called functional fixedness. Functional fixedness was first described by the psychologist Duncker (1945) as the propensity of individuals to be “fixed” on what they believe is the function of an object and to experience difficulty to attribute another function to that object. In the related experiment, human adults were asked to set a candle to a wall in such a way that it could be lit without the wax dripping. The participants were given a limited set of man-made objects: a candle, a box of thumbtacks and a box of matches. To solve the task, they had to put the candle in the emptied box of thumbtacks and then fix the latter to the wall with a thumbtack. Most individuals were unable to use the box of thumbtacks as a probe for the candle if the tacks were contained inside, suggesting that they were functionally fixed on the box’s containing function for the tacks.

Even though both phenomena, mutual exclusivity and functional fixedness, seem to be dependent on the same principle, one tool is linked to one function, they present some specificities that we would like to explore here. Mutual exclusivity has been introduced as a principle that promotes the fast and accurate acquisition of new information under social guidance (Kelemen, 1999; Casler and Kelemen, 2005). Functional fixedness, on the other hand, has been discussed as a phenomenon that actually constrains the flexible restructuring of information due to sticking to functional knowledge that has been previously learnt (German and Defeyter, 2000). We did not find any study mentioning together or even comparing mutual exclusivity and functional fixedness, thus, it is difficult to confirm whether they represent the same phenomenon. As such, we would like to disentangle them and try to understand what are the overlaps and what are their specificities, including here papers from the developmental literature first, and then apply our distinctions on similar studies in non-human species.

The main aims of this review are thus to see (1) whether mutual exclusivity and functional fixedness overlap and what might be their respective specificities and (2) whether mutual exclusivity as an inferential principle could be applied in other domains than language and whether it can be found in non-human species. In order to do that, we will first give an overview of the representative studies of each phenomenon. We will try to analyze the papers on tool use learning that studied one of these phenomena or introduced the terms in order to discuss the results. The tool use literature is very broad and the aim here is not to have an exhaustive review of tool use learning but to pinpoint those studies that showed either mutual exclusivity or functional fixedness and to assess them regarding the inferential mechanisms that help learning the tool function. Based on that, we will identify their specificities, and consequently what might be the overlaps and the dissimilarities of mutual exclusivity and functional fixedness in our view.

After having drawn up the theoretical differences and overlaps of these two phenomena, we will present evidence about both principles in non-human species. We will then give an evolutionary point of view and will summarize the nature of both phenomena.

## Mutual Exclusivity in Psychology

Mutual exclusivity in the domain of psychology is a concept originating from linguistic science. It has been defined as the tendency of humans to attribute exclusively one label to an object when learning a language (Markman and Wachtel, 1988; Merriman and Bowman, 1989). This effect is more commonly found in the earlier stages of life, i.e., from early childhood to childhood (Liittschwager and Markman, 1994; Bion et al., 2013), beginning as early as 16 months of age (Liittschwager and Markman, 1994; Halberda, 2003). Markman and Wachtel (1988) showed that, when confronted with a new label, preschool children (aged from 3 to 5 years old) chose to give this label to an unfamiliar object compared to a familiar one. The results suggest that, starting from the age of 3 years (or even earlier Frank and Poulin-Dubois, 2002; Bion et al., 2013), young children consider that an object is described by one primary label. This propensity to use one label develops with age, as 17-month-old children seem more likely to use such a strategy than 14- and 16-month-old children (Halberda, 2003). Adults also use mutual exclusivity when they face an unfamiliar object with a new label to assign (Golinkoff et al., 1992; Halberda, 2006).

Some authors have argued that this behavior is driven by novelty attraction, where children will more readily associate the new word to the new object (Merriman and Schuster, 1991). However, Golinkoff et al. (1987) showed that mutual exclusivity cannot be explained only by novelty attraction since the authors asked the children to manipulate both familiar and unfamiliar objects to make them equally salient. Although it cannot be excluded that one of the objects remained more familiar than the other despite the manipulation, this protocol might at least have reduced the novelty attraction effect. Furthermore, novelty attraction seems to be the prominent strategy for children up to 2 years old and less for older children (Merriman and Schuster, 1991). For example, Markman and Wachtel (1988) used a control condition where 3–4 years old children faced both a familiar and unfamiliar object. When they were asked to show an object (in this case, there was no label given), the subjects did not show any preference for either type of object and chose at random, suggesting that their choice was not influenced by novelty attraction. An interesting social reasoning alternative has been formulated by Jaswal and Hansen (2006) and Jaswal (2010) who wondered whether children chose the unfamiliar object for an unfamiliar label because of their inferring of the other’s intent as followed: “If the speaker had meant that, s/he would have said that” (Jaswal and Hansen, 2006, p. 163) instead of it being due to children’s inability to give more than one label to an object. In the first case, children would pay more attention to social cues (like pointing and gazing) to choose the object, while in the second situation, they would choose only based on mutual exclusivity. Their studies showed that 2—6-year-old children still chose the unfamiliar object over the familiar one when the adult pointed *or* looked at the familiar object while asking for an unfamiliar label (Jaswal and Hansen, 2006; Jaswal, 2010). However, if the adult asked for an unfamiliar label but pointed *and* looked at the familiar object, the children chose the familiar object (Jaswal, 2010). This suggests that children focused more on their prior label knowledge of the object than on social cues (gazing or pointing) unless both cues were used. Mutual exclusivity seems to not simply be due to social inference alone (what the other wants) but is also a way of organizing knowledge.

These studies suggest that mutual exclusivity in language learning is a strong strategy that cannot be confounded with other phenomena such as novelty attraction or social cues. Although mutual exclusivity is mainly described in language acquisition, its existence in other domains could show that it is not language specific but a general mechanism for efficient learning, including both the acquisition and organization of conceptual knowledge (Markman, 1992; Diesendruck and Markson, 2001; Tomasello, 2001; Casler, 2014).

## Mutual Exclusivity and Functional Fixedness in Tool Use in Humans

Only a small set of studies investigated mutual exclusivity *per se* in tool use in children. Casler and Kelemen (2007) investigated whether children use function mapping for tools (the process of assigning a function to a tool) in a robust way over time. In their study, an adult manipulated two unfamiliar tools in front of a 2-year-old child, then randomly chose one of them to ring some bells inside a box. After the demonstration, the children got to manipulate both tools and insert them in two different boxes, one with the bells inside and another one without the bells in order to control for saliency and affordances. The adult then placed dried pasta on the table with both tools on each side of the pasta, crushed the pasta behind an opaque screen with one of the tools (that the child could not see) and only showed the end result of the crushed pasta. When presented with both tools and the pasta, children used the same tool that was demonstrated earlier (for ringing the bells inside the box) for this new goal. Thus, this study suggests that 2-year-old children did not show mutual exclusivity for tools yet as they used the same tool for two different functions. Nevertheless, from 3 years of age children chose the demonstrated tool when asked to use it for a purpose that was similar to the demonstrated goal, yet avoided using that tool when asked to perform a different function (Casler and Kelemen, 2005). Based on this, the authors concluded that children considered tools as made for that particular purpose and dedicated for that after only one exposure to a demonstration. Using the same method as Casler and Kelemen (2007), in a recent study, Petõ et al. (2018) showed 4-year-old children a demonstration of a novel tool being used for a specific function (e.g., turning on a lamp), either by an in-group demonstrator speaking the children’s native language or by an out-group demonstrator speaking a foreign language. Then in the testing phase, the children had the choice between the tool from the demonstration phase and a novel tool to achieve a new goal (e.g., crushing plasticine). The results showed that children preferentially chose the new tool over the old one for the new goal in the in-group condition but not in the out-group condition. This shows the propensity of children for assigning tools to a specific function and thus, to learn from in-group members compared to out-group members.

Mutual exclusivity in tool use seems to develop around 3 or 4 years of age and is dependent on the social context. In the light of the above results, there are two conditions, similarly to the predictions of the same principle in the language domain, that must be fulfilled for mutual exclusivity in tool use: (1) the same function has to always be reached by the same tool (like the same object should be named by an already known word, if possible); (2) this same tool cannot be used for other functions than this one (like the same word cannot refer to different kinds of objects, or meanings). These aspects of functional knowledge are assumed to be shared, like the meaning of words, by members of the cultural community.

The propensity to fixate one function to one object, called functional fixedness, also exists in older children and adults (12—25-year-olds), and is present in technological and non-technological societies as well (e.g., the Shuar population of Ecuador; German and Barrett, 2005). In a series of experiments, German and Defeyter (2000) and German and Barrett (2005) showed that 6–7-year-old children but not 5-year-olds had difficulties using an object in a distorted way in order to attain a novel goal (e.g., build a tower from objects otherwise used as containers for reaching an object or build a bridge using a spoon). Interestingly, younger children (5-year-olds) in contrast to older children performed better under conditions where they had been exposed to the functional use of the objects (Defeyter and German, 2003). Correspondingly, a study with 4-year-olds showed the lack of functional fixedness after an adult’s demonstration of the objects’ function (Nielsen, 2012), showing that in younger children the reinstatement of past experiences has a smaller impact on their problem solving.

In adults, this fixation effect became stronger when primed before the test (i.e., being told the main function of the object) in a creativity task although subjects seemingly tried to overcome the creativity inhibition past the first attempts (Camarda et al., 2018; Munoz-Rubke et al., 2018). Additional EEG data suggests that the participants who were primed might have relied on top-down control to overcome the fixation as they all showed higher alpha power (related to the activation of top-down processes) regardless of each participant’s level of functional fixedness (Camarda et al., 2018). Interestingly, regardless of the learning modality (reading, watching the tool’s function or physical manipulation of the tool), adults showed the same level of functional fixedness in puzzle tasks (Munoz-Rubke et al., 2018) and they had more difficulties to find other uses for objects rated with high functional fixedness (i.e., objects usually restricted to their common use, such as a desktop) as opposed to those with lower functional fixedness (such as a blanket) in an imagined survival scenario (Kroneisen et al., 2021).

This phenomenon looks very robust already from its onset, because even prior experience with the affordance properties of objects as tools does not seem to change children’s behavior, namely, does not result in applying those properties in order to solve novel problems. For example, 6-year-old children had difficulties using a bucket to pour water inside a tube in order to retrieve a ball. Furthermore, being exposed to the affordances of the bucket (using a transparent bucket and putting it closer to the tube or demonstrating the target action) did not change children’s performance. However, 7- and 8-year-olds showed higher flexibility regarding the use of the bucket as a water container, a pattern that emerges from their ability to discard the unsuccessful fixed strategy that they had also tried and search for alternative solutions. Therefore, their flexibility is rather a proof of their emergent innovation capacities and not of their resistance to functional fixedness (Hanus et al., 2011; Ebel et al., 2020). Taken together, these results suggest that functional fixedness appears around 6 years of age and develops through adulthood and that it may be a strong phenomenon that exists in more than one culture.

Even though mutual exclusivity and functional fixedness were not compared in a single study with similar methodology, they seem to develop at different times in childhood, with mutual exclusivity appearing earlier than functional fixedness. The earlier emergence of mutual exclusivity could be explained by its role in acquisition: the main function of this principle is the guidance of mapping the common meanings, being it the lexicon of a language, or the nest of functional knowledge. As a clue for establishing common knowledge, the application of this inferential rule is induced by social partners, the sources of shared knowledge. Consequently, the learning process itself leads the organization of information into conventional forms that are shared by partners in the community. This principle of contrast, as described, implies the possibility to be used in general to several domains of knowledge. Besides, mutual exclusivity seems to become weaker with age, while functional fixedness remains a strong cognitive constraint, even later in adulthood (Duncker, 1945; German and Defeyter, 2000), underlining the specific function of mutual exclusivity in the process of learning.

Mutual exclusivity appears to be a flexible strategy. Three-year-old children tend to assign a new label to the whole object when it is unfamiliar. They are, however, able to adapt and tend to find which part of the object to assign this new label to, when the object is already labeled (Markman and Wachtel, 1988). Moreover, Liittschwager and Markman (1994) showed that children can learn second labels for familiar objects under certain conditions like contradictory information or an overload of information. In the domain of tool use, individuals also understand that an object may have more than one function, even if they still believe that the object has one main function for which it was designed (Casler and Kelemen, 2005). As empirical evidence, in Petõ et al. (2018) paper, children displayed mutually exclusive choices after watching demonstrators speaking their native language, however, they were able to use the same tools for different purposes when they observed a demonstrator speaking a foreign language. In this paper the authors argued that foreign language could signal to the children that they did not come from the same cultural background as the demonstrator, and thus they might not have shared the same knowledge about tools and their functions. The authors proposed that children understand that different tools can serve the same purpose depending on one’s cultural background. Mutual exclusivity seems to be context-dependent and can further be refined, even when the individuals are facing a culturally familiar context.

On the other hand, functional fixedness seems to be less flexible. Learning about a tool as made for a specific function makes it possible to apply this knowledge in a variety of situations, for similar purposes and makes it possible to form expectations for others in order to solve similar problems as well. This benefit in fast mapping could result in some disadvantages as well: the habitual application of this knowledge would result in difficulty to override it when necessary. Humans (among numerous other species) show a general tendency to approach problems in a certain way, using an already learnt motor pattern or a cognitive strategy as a result of experience and habits (Logan, 1985; Cañas et al., 2003; von Bastian and Druey, 2017). If individuals are used to doing a task in a certain way, they have a harder time trying to do it another way. Thus, while functional fixedness as a cognitive constraint could enhance looking faster for a familiar needed tool, it would also weaken the possibility to flexibly turn to alternative uses of the same object.

Some studies suggest that experts tend to be more cognitively rigid in their tasks than beginners, which is why they are efficient in their work (Anzai and Yokoyama, 1984; Frensch and Sternberg, 1989) yet this does not mean that experts always remain inflexible. This tendency starts early in infancy: 12–18-month-old infants are fixed on their use of familiar tools such as a spoon. For example, they have difficulties grasping a spoon by the bowl part and inserting the handle into a box to solve a task compared to the use of an unfamiliar tool (Barrett et al., 2007; Kaur et al., 2020). Overall prior experience in tool use seems to make individuals less flexible and less variable in their actions with tools (Barrett et al., 2007). However, despite this rigidity some studies have shown that functional fixedness can be overridden, with the help of different strategies such as decomposing the object into different components and thus, little by little getting a clearer idea about the shape, functions, etc. of each part of the object (McCaffrey, 2012) or training to change the representation of an otherwise fixed representation of a problem (Patrick and Ahmed, 2014).

Furthermore, one of the main divergent points between mutual exclusivity and functional fixedness in our view is related to the social context of their induction. Learning in children, particularly social learning, is tightly linked to the social cues in the environment and the communicative skills of the social partners that demonstrate the target actions (as emphasized by natural pedagogy theory, see Gergely and Csibra, 2013). Mutual exclusivity as an inferential principle is triggered by social partners, and is dependent on an appropriate social learning situation, while functional fixedness seems to be a byproduct of the learning process, a cognitive constraint, generalized across cultures and thus observable independently of the social context. Functional fixedness, indeed, could originate from the expectation shaped by social sources, namely what is the main dedicated function of the object, but the experience with a specific object, the repeated use of it for the same purpose contributes largely to the phenomenon.

What about functional fixedness and mutual exclusivity in other species? We will explore similarities and dissimilarities of mutual exclusivity and functional fixedness in the non-human literature, with the objective to investigate whether this inferential rule is used for learning new information fast or/and whether it constrains the flexible refinement of acquired knowledge.

## Mutual Exclusivity and Functional Fixedness in Symbol and Tool Use Acquisition in Other Species

Non-human species [rhesus monkeys (*Macaca mulatta*), dogs (*Canis lupus familiaris*), pigeons (*Columba livia*), sea lions (*Zalophus californianus*)] seem capable of mapping new associations by emergent matching; they perform new conditional discriminations without explicit training after being exposed to a non-social associative stimulus-stimulus matching task (Kastak and Schusterman, 2002; Aust et al., 2008; Gazes et al., 2018). However, the findings underline that non-human participants do not show *learning* by exclusion, since similar learning rates were observed under exclusion with trial and error tests as in a control trial and error test alone (Gazes et al., 2018).

Only a few studies tested symbol acquisition by mutual exclusivity in non-human species within a social learning context. This is probably because such studies require human vocabulary understanding or even human protolanguage production (Pepperberg and Wilcox, 2000). Furthermore, it requires training the subjects to associate human words with different objects, which is difficult and time-consuming. Another example of mutual exclusivity in symbol acquisition is Rico, a dog who possessed an impressive vocabulary of over 200 different human words, each word being assigned to a unique object (Kaminski et al., 2004). Thanks to that, Rico was able to fetch the correct objects whenever a human gave him the instruction. The experimental setup was similar to those in psycholinguistics: the dog faced a familiar object (whose label was known to Rico) and a novel object, then was told to bring an object with an unknown label. Rico seemed to infer that the experimenter was referring to the novel object, because the familiar object already had another label (similar results have been found with another dog; Pilley and Reid, 2011). Mutual exclusivity was also hypothesized to be the reason why African gray parrots (*Psittacus erithacus*) had a hard time associating labeled objects with colors, unless both names were taught while being paired together, such as “blue square” instead of “blue” and “square” heard separately (Pepperberg and Wilcox, 2000). Another study (Beran, 2010) also seemed to show mutual exclusivity with a female chimpanzee (*Pan troglodytes*) in a matching-to-sample test, where she had to associate either lexigrams or photographs with English words. Whenever the word was unknown to her, she avoided associating it with a known stimulus and instead chose the unfamiliar one. Such studies suggest that those non-human animals used a cognitive strategy similar to mutual exclusivity in symbol learning.

Despite the broad interest in the investigation of tool use and its acquisition in non-human species showing some extraordinary capacity in this respect in New Caledonian crows (*Corvus moneduloides*) (Rutz and St Clair, 2012; Taylor et al., 2012; McGrew, 2013; Jelbert et al., 2018) and some apes (Byrne and Russon, 1998; Yamamoto et al., 2013; Gruber et al., 2015), these studies do not focus on mutual exclusivity and functional fixedness as potential mechanisms by which the tool use capacity is formed. Only one study investigated functional fixedness *per se* (Ebel et al., 2021). Others use this term to discuss some of the observed behavior (Gruber et al., 2011; Hanus et al., 2011). We will describe those studies here and show how individuals’ behavior can be interpreted in terms of mutual exclusivity and/or functional fixedness. Because tool use can be hard to define in non-human species, we will use here the following definition (St Amant and Horton, 2008, p. 1203): “Behaviors aimed at altering a target object by mechanical means and behaviors that mediate the flow of information between the tool user and the environment or other organisms in the environment.”

In the study that investigated functional fixedness, four species of captive non-human primates [bonobos (*Pan paniscus*), chimpanzees (*Pan troglodytes*), gorillas (*Gorilla gorilla*), and orangutans (*Pongo abelii*)] showed functional fixedness and an inability to use objects in a different manner than the one they were primed to Ebel et al. (2021). For example, individuals who had been fed with grissini before the experiment and therefore experienced the grissini as food showed less occurrences of using the grissini as a tool to rake in grapes compared to naive individuals who had no such prior experience (Ebel et al., 2021).

Functional fixedness was also introduced to explain captive chimpanzees’ behavior in a “floating object” task (mentioned earlier as a type of test with children) where chimpanzees had to retrieve a peanut from a tube by pouring water into it using their water dispenser (Hanus et al., 2011). In this experiment, chimpanzees had a hard time solving the task with their familiar water dispenser (by collecting the water with their mouth then spitting it in the tube) and improved their performances when the water dispenser was replaced by a novel one with different color and appearance. Thus, it seems that chimpanzees fixated one function to one tool and could not use the object in a different way. Such behavior was also observed in captive chimpanzees (Harrison and Whiten, 2018) and capuchin monkeys (*Cebus apella*) (Renner et al., 2017) who were unable to use materials from their daily life (nest materials, drinking or bathing) in alternative ways. Interestingly, fixation to a functional use also develops when seeing a conspecific using the tool in a specific way (Whiten et al., 2005). Introducing an alternative way of using a foraging tool in two wild chimpanzee communities was also not enough to prompt these communities to have an insight based on the affordance properties of the object and adopt a new functional use of it (use of a leafy stick as a dipping stick or a sponge to get honey) (Gruber et al., 2011). All those studies seem to highlight that prior experience may have an impact on non-human primates’ use of tools, thus leading to functional fixedness in tool use development.

In sum, there is evidence –yet quite sparse– in support that non-human species are equipped with the inferential principle of mutual exclusivity that helps them in symbol learning. In problem solving scenarios, the inclination of sticking to an already exercised function of an object is apparent in non-human species, especially in chimpanzees. The question remains, though, whether any non-human species could apply mutual exclusivity for other learning purposes apart from human directed symbol acquisition.

## An Evolutionary View of Mutual Exclusivity and Functional Fixedness

A common hypothesis for the function of mutual exclusivity is that it helps language acquisition, just as many other lexical constraints (Golinkoff et al., 1992; Liittschwager and Markman, 1994; Halberda, 2006). Undeniably, individuals may face situations where there could be too much information, making it difficult to know which category the word refers to. In such scenarios, mutual exclusivity is used as a fast way to narrow down the possibilities of the meaning of the words (Markman et al., 2003). With respect to tool use, the advantages would be similar to language acquisition, which is the application of a strategy to learn new tools’ function (fast mapping). It is especially useful for children, as they discover a lot of artifacts around them, yet they do not have all the information needed to know their function (Petõ et al., 2018).

For functional fixedness, authors have hypothesized that it is a constraint that serves as a barrier to innovation (Brosnan and Hopper, 2014; Carr et al., 2016; Gruber, 2016). This could seem contradictory with evolution and the advantages innovation brings to the social group (more productivity, exploitation of novel resources or adaptation to changing environments (Reader and Laland, 2003; Brosnan and Hopper, 2014). However, such advantages could be quite limited as well (Reader and Laland, 2003; Brosnan and Hopper, 2014). In a stable environment, individuals do not necessarily need innovations since chances are high that they are already well adapted to their environment (Brosnan and Hopper, 2014). If a novel behavior is created (for example in tool use), there are chances that this novelty may not be as adaptive as the old behavior, therefore not making it worthy to keep. This could be one of the reasons why innovation is existent but less frequent in nature (Reader and Laland, 2003; Brosnan and Hopper, 2014; Reader et al., 2016), thus explaining the existence of cognitive biases such as conservatism (Hrubesch et al., 2009), conformity (Boyd and Richerson, 1985), or functional fixedness (Brosnan and Hopper, 2014; Carr et al., 2016). Furthermore, human societies are very technology-oriented [the term of *technology* here is used as its more global definition of “the methods, systems, and devices which are the result of scientific knowledge being used for practical purposes” (Collins Dictionary), not just of industrial techniques]. The development of such an artifact-filled environment has probably been made possible thanks to the way humans heavily rely on specialized tools (Casler and Kelemen, 2005), thus it would be more effective to keep using those tools without much innovation (Casler and Kelemen, 2005).

As for non-human species, we have seen that mutual exclusivity might also exist for them. Considering the lack of studies on the matter, it is still uncertain whether mutual exclusivity can be found as much in symbol learning as it does in humans. However, we can hypothesize that the advantages would be similar for non-human species as for humans, on the grounds that researchers observed mutual exclusivity when teaching human words to non-human animals (Pepperberg and Wilcox, 2000). As for tool use, a specialization for tools and a fixation on one function have been observed in some species such as in chimpanzees. Indeed, some chimpanzee communities possess tool sets specialized for a certain goal (ant/termite fishing, honey extraction), and each tool of the sets is used for one adapted step of the activity (Sanz and Morgan, 2007; Schöning et al., 2008; Sanz et al., 2010; McLennan, 2011; Hashimoto et al., 2015). For example, one tool is used to puncture a hole in the ant/termite nest, then another one is used to fish them out. Each of these tools is physically different from the other tools of the set. Some studies also show that army-ant-eating may vary culturally among chimpanzee communities (Schöning et al., 2008). This suggests that those chimpanzees also might view each of those tools as having a specific function, similarly to humans. Observations from different studies suggest that those tool sets are community-specific, which would explain why it is not ubiquitous to a same subspecies of chimpanzees (Schöning et al., 2008; Furuichi et al., 2015; Hashimoto et al., 2015). Tool specialization can be found rarely among few non-human animals, such as chimpanzees and its different subspecies (Sanz and Morgan, 2007; Schöning et al., 2008; Sanz et al., 2010; McLennan, 2011; Yamamoto et al., 2013; Hashimoto et al., 2015). Because the tools are specific for the tasks, much thought is given to the choice of the tools. Such tool specialization could be one of the bases for explaining why individuals fixate on a particular tool for a specific function.

## Conclusion

The first aim of this review was to explore whether mutual exclusivity and functional fixedness overlap and what might be their respective specificities. Although mutual exclusivity and functional fixedness share and depend on the same principle of “one tool is linked to one function,” they clearly vary in the roles they play in learning and cognition. Functional fixedness appears to be a cognitive constraint that stems from prior experience (German and Defeyter, 2000; Defeyter and German, 2003; German and Barrett, 2005) and thus, constrains the individuals’ reasoning. Meanwhile, mutual exclusivity appears to be an inferential process that guides fast acquisition of new information that not only comes from a partner but is also shared within a social community (Casler and Kelemen, 2007; Petõ et al., 2018). The main characteristics that underlie these differences are their divergence in flexibility and their variation in dependence on the social context: mutual exclusivity being flexible, while dependent on social learning, and functional fixedness being more of a habitual cognitive constraint and less dependent on social context.

The second aim of the review was to investigate whether mutual exclusivity as an inferential principle could be applied in other domains than language and whether it could be found in non-human species as well. Mutual exclusivity is mostly known and applied in language acquisition (Markman and Wachtel, 1988; Merriman and Bowman, 1989), but, as recent research revealed, can also be applied in function learning. This review highlights that humans, and especially children, use mutual exclusivity as a strategy not only for learning a language, but also for learning tools’ function. Indeed, based on the summary of relevant research, it is argued that mutual exclusivity does not simply guide label learning or function mapping, but by implying contrast and relational information as well, it also contributes to organizing knowledge. Furthermore, in language/symbol acquisition as well as in tool function learning, mutual exclusivity does not seem to be limited to humans, as some studies show occurrences of a similar mechanism in other species (mainly in non-human primates). It would thus be interesting to have more studies in the future that focus on mutual exclusivity in order to better understand its origin and effects in other species.

## Author Contributions

TT, RE, DB, and IK contributed to the conception of this review. TT drafted and wrote the manuscript. DB, RE, and IK critically revised the manuscript and gave final approval of the submission of this review. All authors contributed to the article and approved the submitted version.

## Conflict of Interest

The authors declare that the research was conducted in the absence of any commercial or financial relationships that could be construed as a potential conflict of interest.

## Publisher’s Note

All claims expressed in this article are solely those of the authors and do not necessarily represent those of their affiliated organizations, or those of the publisher, the editors and the reviewers. Any product that may be evaluated in this article, or claim that may be made by its manufacturer, is not guaranteed or endorsed by the publisher.
